# MASH: the nexus of metabolism, inflammation, and fibrosis

**DOI:** 10.1172/JCI186420

**Published:** 2025-09-16

**Authors:** Gregory R. Steinberg, Andre C. Carpentier, Dongdong Wang

**Affiliations:** 1 Centre for Metabolism, Obesity and Diabetes Research, Division of Endocrinology and Metabolism, Department of Medicine, Faculty of Health Sciences, McMaster University, Hamilton, Ontario, Canada.; 2 Centre de recherche du Centre hospitalier universitaire de Sherbrooke, Department of Medicine, Université de Sherbrooke, Sherbrooke, Quebec, Canada.

## Abstract

Metabolic dysfunction–associated steatohepatitis (MASH) is a progressive form of liver disease characterized by hepatocyte injury, inflammation, and fibrosis. The transition from metabolic dysfunction–associated steatotic liver disease (MASLD) to MASH is driven by the accumulation of toxic lipid and metabolic intermediates resulting from increased hepatic uptake of fatty acids, elevated de novo lipogenesis, and impaired mitochondrial oxidation. These changes promote hepatocyte stress and cell death, activate macrophages, and induce a fibrogenic phenotype in hepatic stellate cells (HSCs). Key metabolites, including saturated fatty acids, free cholesterol, ceramides, lactate, and succinate, act as paracrine signals that reinforce inflammatory and fibrotic responses across multiple liver cell types. Crosstalk between hepatocytes, macrophages, and HSCs, along with spatial shifts in mitochondrial activity, creates a feed-forward cycle of immune activation and tissue remodeling. Systemic inputs, such as insulin-resistant adipose tissue and impaired clearance of dietary lipids and branched-chain amino acids, further contribute to liver injury. Together, these pathways establish a metabolically driven network linking nutrient excess to chronic liver inflammation and fibrosis. This Review outlines how coordinated disruptions in lipid metabolism and intercellular signaling drive MASH pathogenesis and provides a framework for understanding disease progression across tissue and cellular compartments.

## Introduction

Metabolic dysfunction–associated steatotic liver disease (MASLD) is defined as the presence of excess hepatic triglycerides on imaging (for example, >5.6% volume using proton density fat fraction with magnetic resonance spectroscopy) or histology with at least one feature of the metabolic syndrome, including obesity or type 2 diabetes in the absence of excess alcohol intake or other chronic liver disease (1). This condition affects a third of the global adult population and up to 70% of individuals living with type 2 diabetes (1). It is a silent pandemic; as few as 5% of the individuals affected are aware of the disease (2), and there is considerable clinical inertia with regard to diagnosis and management among non-hepatologists (3). Hepatic inflammation and fibrosis develop in approximately 20%–30% of people with MASLD, a condition called metabolic dysfunction–associated steatohepatitis (MASH), and this leads to a dramatic increase in morbidities and mortalities including liver failure, hepatocarcinoma, cardiovascular diseases, cognitive decline, and chronic kidney diseases (1). Therefore, understanding the mechanisms that lead to the transition from MASLD to MASH is critical to identify new therapeutic targets that may maximize therapeutic benefits.

**Figure 1:**
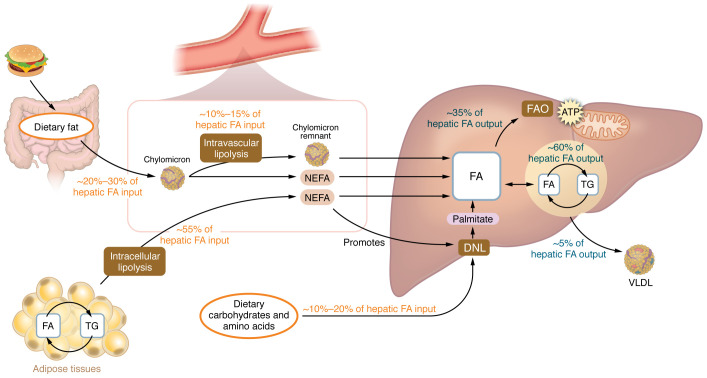
Interrelated mechanisms of intrahepatic triglyceride accretion in MASLD. Interconnections between fatty acid (FA) delivery from dietary sources, including chylomicrons and nonesterified fatty acids (NEFA) from adipose tissue, and intravascular lipolysis. This process intersects with liver-specific effects related to de novo lipogenesis (DNL) from carbohydrates, including fructose and amino acids, fatty acid oxidation (FAO), and very low-density lipoprotein (VLDL) production. TG, triglycerides.

Hepatocytes, hepatic stellate cells (HSCs), and liver macrophages operate in a finely tuned and spatially coordinated network to support metabolic homeostasis (4). In the healthy liver, changes in hepatocellular lipid acquisition from fatty acid uptake and de novo lipogenesis (DNL) are balanced with lipid consumption and storage from fatty acid oxidation and very low-density lipoprotein (VLDL) export, respectively, over fasting and feeding periods throughout the day (Figure 1). Hepatocytes, comprising about 70%–80% of liver volume, carry out critical metabolic functions including gluconeogenesis and lipid and cholesterol metabolism. Kupffer cells, the resident embryonically derived liver macrophages, continuously sample portal blood for microbial components and debris, playing a crucial role in immune surveillance and clearance of gut-derived antigens (5, 6). They also contribute to immune tolerance and support hepatocyte function through clearance of senescent cells and orchestration of early responses to tissue injury (6). Macrophages and hepatocytes interact closely with HSCs, which, in addition to vitamin A storage, play several essential roles in maintaining liver structure and function by regulating sinusoidal blood flow through contractile responses and contributing to the homeostatic turnover of extracellular matrix (ECM) components (7, 8). Under most homeostatic conditions, hepatocytes, Kupffer cells, and HSCs primarily rely on mitochondrial oxidative phosphorylation and fatty acid metabolism to support their physiological roles (4–6).

The stability of this homeostatic network is highly susceptible to chronic metabolic perturbation, and in the context of sustained overnutrition, hepatocyte lipid handling becomes dysregulated. This results in the accumulation of triglycerides and, more importantly, toxic lipid and metabolic intermediates that can be primarily attributed to defects in four interrelated pathways: (a) increased uptake of fatty acids from extrahepatic sources including diet and adipose tissue; (b) increased DNL; (c) reduced fatty acid oxidation; and (d) impaired VLDL production. While recent studies have highlighted how VLDL production impacts the risk of liver damage versus cardiovascular complications in people with MASLD (9), we will not focus on this topic, in order to concentrate on interactions with the first three variables. Importantly, with the accumulation of lipotoxic and metabolic intermediates in the liver, there is recruitment and differentiation of bone marrow–derived monocytes that give rise to monocyte-derived macrophages (10–13), which express TREM2 and are commonly referred to as lipid-associated macrophages (14), and/or scar-associated macrophages (15). Steatotic hepatocytes and activated immune cells also trigger a phenotypic shift in HSCs marked by loss of lipid droplets and increased glycolysis, leading to proliferation and transdifferentiation into fibrogenic myofibroblasts that are characterized by a contractile, ECM-producing phenotype (4–6). These changes in hepatocyte, macrophage, and HSC identity and function reinforce a feed-forward cycle of metabolic stress, inflammation, and tissue remodeling that characterize the development of MASH and fibrosis.

In this Review we aim to describe in an integrative manner how the metabolic pathways of fatty acid uptake, DNL, and fatty acid oxidation are tightly interconnected and not only contribute to hepatic steatosis but also generate metabolic intermediates that activate HSCs and macrophages to promote MASH and fibrosis.

## Uptake of fatty acids from extrahepatic sources

### Increased uptake of dietary fatty acids.

After a meal, triglycerides are packaged into chylomicrons by the intestine and hydrolyzed by lipoprotein lipase (LPL), delivering fatty acids to muscle and adipose tissue. Hepatic dietary fatty acid uptake (from chylomicron remnants plus chylomicron-triglyceride non-esterified fatty acid spillover) accounts for 20%–30% of total fatty acids taken up by the liver after meals (16) (Figure 1). A meta-analysis of large genome-wide association studies has linked MASLD with genetic polymorphisms associated with lower activity of adipose tissue LPL, an enzyme that is not normally expressed in hepatocytes (17). This is in accordance with high risk of MASLD independent of obesity in subjects with LPL deficiency (18). It is important to note that not all dietary fats are equivalent, as intake of saturated fatty acids, particularly palmitate and stearate, is much more closely linked to MASLD than intake of polyunsaturated fatty acids (19, 20). Consistent with these associations, spatial lipidomics has found that saturated fatty acids are enriched within fibrotic areas while polyunsaturated fatty acids are reduced (21). And although saturated fatty acids can promote inflammatory pathways, this likely does not involve direct binding to Toll-like receptor 4 (TLR4) (22). Instead, saturated fatty acids may promote inflammation and fibrosis in MASH by increasing free cholesterol (23).

**Figure 2:**
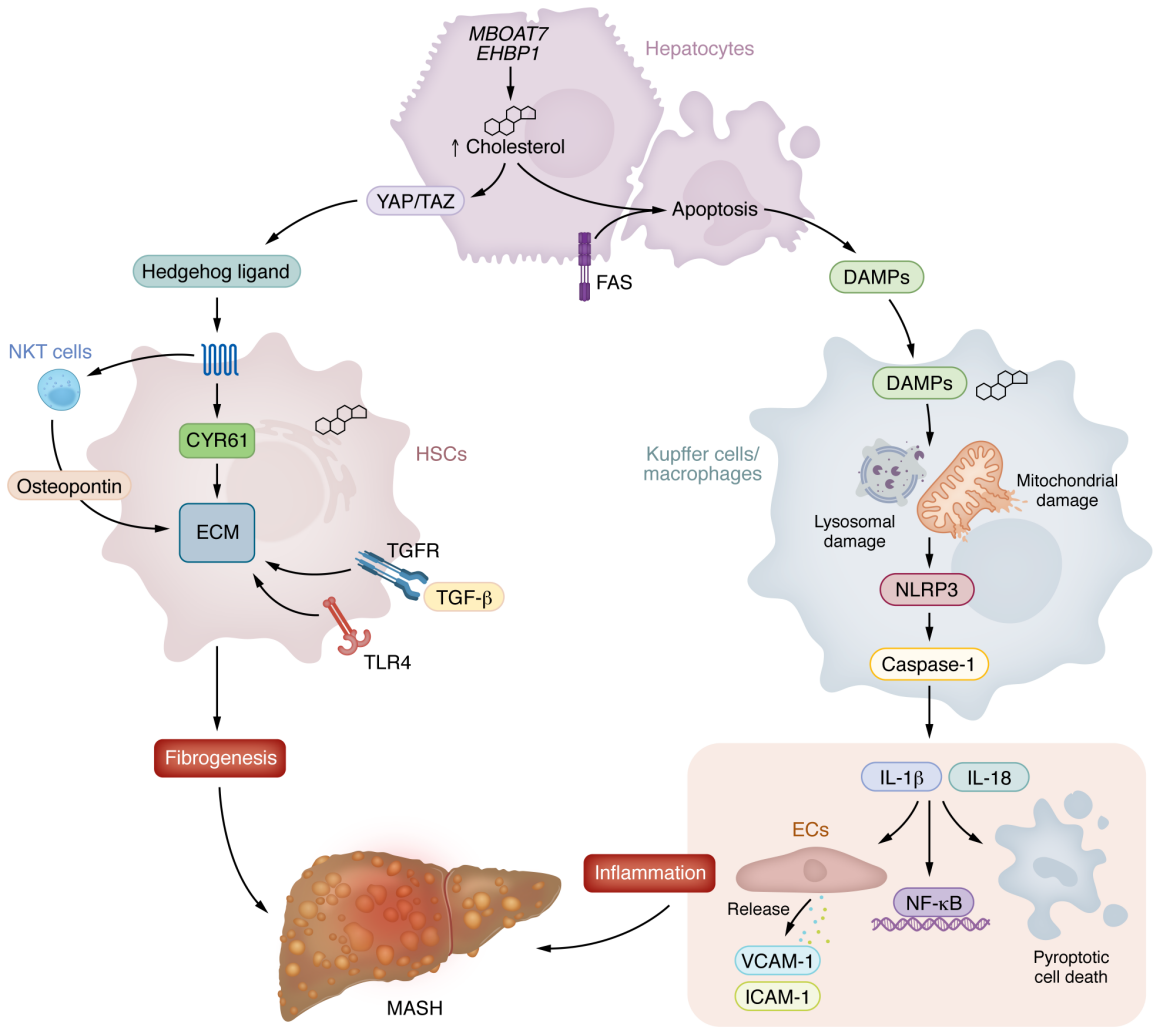
Increases in free cholesterol trigger liver inflammation and fibrosis. (Left) Loss-of-function polymorphisms in genes such as *MBOAT7* and *EHBP1* lead to increased free cholesterol levels and activation of the YAP/TAZ pathway. Cholesterol enhances hepatic stellate cell (HSC) responsiveness to TGF-β via TLR4/TGF receptor and stabilizes YAP/TAZ signaling in hepatocytes, promoting the secretion of Indian hedgehog ligands, activation of NKT cells, HSC activation through communication network factor 1 (CYR61) and osteopontin, and the production of fibrogenic ECM. (Right) Cholesterol accumulation in hepatocytes and macrophages promotes cell death and inflammation by enhancing sensitivity to TNF and FAS ligands, disrupting mitochondrial membranes, and inducing phagolysosomal damage, which together trigger NLRP3 inflammasome activation in macrophages causing the release of IL-1β and IL-18. These cytokines activate NF-κB signaling and upregulate chemokines and adhesion molecules, further driving immune cell recruitment and amplifying liver inflammation.

In livers of both mice and humans with MASH, there are increases in free cholesterol in hepatocytes, Kupffer cells, and HSCs (24–28) (Figure 2). In hepatocytes, free cholesterol increases sensitivity to tumor necrosis factor– (TNF) and Fas (CD95)-mediated apoptosis, resulting in the release of cholesterol crystals and other danger-associated molecular patterns, such as endogenous ATP and uric acid (29–31). Increases in free cholesterol disrupt mitochondrial membrane integrity, impairing respiratory capacity and inducing mitochondrial damage, which promotes the release of mitochondrial DNA (mtDNA) (32), while cholesterol sequestration within lysosomes induces phagolysosomal damage (33) that leads to the release of cathepsins (34, 35). The release of cholesterol crystals, ATP, uric acid, mtDNA, and cathepsins triggers activation of the NLRP3 inflammasome, a cytosolic multiprotein complex that is highly expressed in macrophages compared with hepatocytes. This leads to the cleavage of pro-caspase-1 into active caspase-1, which subsequently processes IL-1β and IL-18 into their mature forms (34–36). IL-1β and IL-18 receptor binding in hepatocytes promotes pyroptotic cell death and activates NF-κB, establishing a self-amplifying loop of proinflammatory signaling that also induces chemokines (e.g., CXCL8) and adhesion molecules (e.g., ICAM-1, VCAM-1), promoting further recruitment of neutrophils and monocytes (37, 38). This cascade culminates in Kupffer cell activation and the formation of hepatic crown-like structures surrounding necrotic hepatocytes (27, 28). Treatment with a selective NLRP3 inhibitor (MCC950) was found to reduce liver fibrosis in the methionine- and choline-deficient model of MASH (26). However, when mice were fed a high-fat and high-cholesterol diet, pharmacological inhibition of the NLRP3 inflammasome with MCC950 or genetic inhibition of NLRP3 or caspase-1 did not affect fibrosis (39). These data suggest that NLRP3 inflammasome inhibitors may be more effective in the context of advanced fibrosis and cirrhosis than MASH, or alternatively, it may be necessary to combine these compounds with metabolically based therapies that reduce steatosis. Whether NLRP3 inflammasome inhibitors will be safe and effective in people is the focus of several ongoing phase I/IIa clinical trials.

Cholesterol also plays a crucial role in HSC activation by amplifying profibrotic signaling pathways (Figure 2). Quiescent HSCs store cholesterol in an esterified form to prevent toxicity; however, free cholesterol accumulation in HSCs enhances fibrogenic activation by disrupting membrane integrity, altering intracellular signaling, and sensitizing HSCs to profibrotic stimuli through multiple pathways. One mechanism involves cholesterol-driven TLR4 activation, which enhances HSC responsiveness to TGF-β by downregulating its inhibitory receptor BAMBI (25, 40). Cholesterol also stabilizes Yes-associated protein/transcriptional coactivator with PDZ-binding motif (YAP/TAZ) in hepatocytes (41, 42) through a soluble adenylyl cyclase/calcium/RhoA–mediated pathway (43), which promotes HSC activation through the secretion of communication network factor 1 (CYR61) (44). YAP/TAZ activation also promotes the secretion of Indian hedgehog ligand, which further propagates HSC activation and promotes ECM production (45, 46). These effects are further perpetuated as cholesterol accumulation enhances mechanotransduction pathways, stabilizing YAP/TAZ signaling, which reinforces HSC contractility and ECM remodeling (47). The activation of the Hedgehog pathway is also associated with activation of type I natural killer T cells and increases in osteopontin, which further accelerates liver fibrosis in mice (48, 49). Importantly, recent studies have found that common loss-of-function polymorphisms linked to MASH, including *MBOAT7* (50) and *EHBP1* (51), lead to increases in free cholesterol and activation of the YAP/TAZ pathway, independently of differences in steatosis (Figure 2). As targeted inhibition of hepatocyte TAZ (52, 53) or HSC YAP (54) has been shown to reverse fibrosis in mouse models, these data suggest that individuals with these polymorphisms or high levels of circulating Indian hedgehog ligand may be particularly responsive to potential therapies inhibiting the YAP/TAZ pathway.

Together these data suggest that in MASH, increased circulating dietary fatty acids from impaired adipose tissue clearance of chylomicron remnants may increase saturated fatty acids, which promote accumulation of free cholesterol that drives proinflammatory and profibrotic activation of hepatic macrophages and HSCs, creating a feed-forward cycle of immune activation and fibrogenesis that accelerates MASH progression.

## White and brown adipose tissue insulin resistance

**Figure 3:**
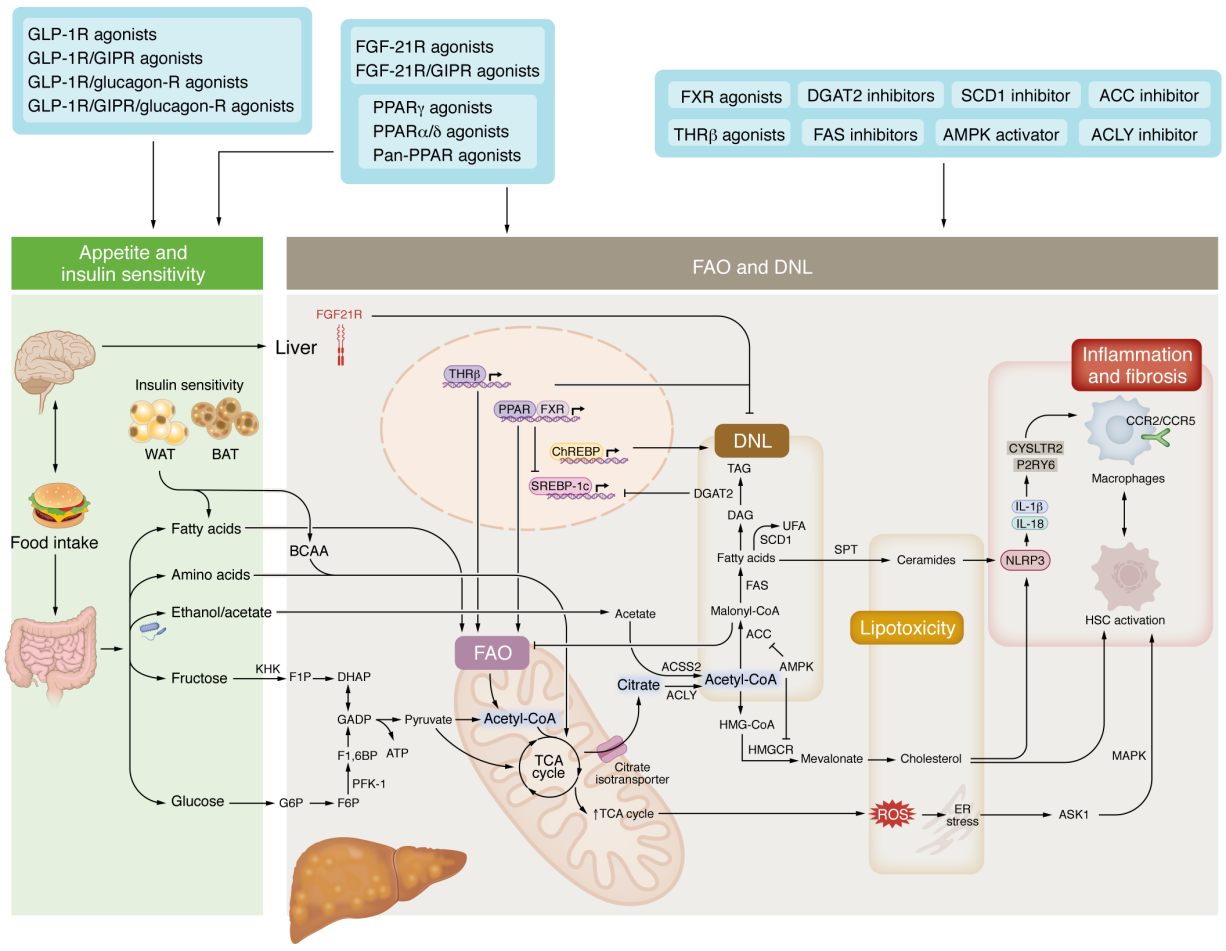
Targets in pathways contributing to elevated DNL and ceramide production in MASH. Delivery of diet-derived FAs, amino acids, fructose, and glucose to the liver supplies carbon for hepatic DNL. Both white and brown adipose tissues function as metabolic sinks for FAs and BCAAs, which also contribute carbon to DNL. GLP -1R agonists reduce food intake via central mechanisms and improve adipose tissue insulin sensitivity, reducing steatosis and MASH. DNL is initiated when excess substrate availability via FAO, carbohydrates, and amino acids converges at the TCA cycle and increases mitochondrial citrate levels. The citrate isotransporter (SLC25A1) exports excess citrate to the cytosol, where it is converted to acetyl-CoA by ATP-citrate lyase (ACLY). Gut microbiome–derived ethanol can also contribute to the hepatic acetyl-CoA pool via ACSS2. Acetyl-CoA may translocate to the nucleus and modulate gene expression programs related to DNL. Nuclear receptors such as FXR, THRβ, and PPAR also regulate gene expression to inhibit DNL, promote FAO, and suppress transcriptional regulators, including ChREBP and SREBP-1c, which both drive expression of DNL enzymes. FXR, THRβ, and PPAR agonists improve MASH by simultaneously inhibiting DNL and enhancing FAO. Acetyl-CoA is also a precursor for cholesterol synthesis, a process closely linked to liver inflammation and fibrosis. Most cytosolic acetyl-CoA is converted to malonyl-CoA and then to FAs. Targeting key nodes in this pathway — including inhibition of the citrate isotransporter, ACLY, ACC, or FAS or activation of AMPK — suppresses DNL and ameliorates hepatic steatosis. Additionally, inhibition of DGAT2 and activation of SCD1 reduces triglyceride synthesis, thereby attenuating DNL and improving MASH. Ceramides derived from FAs activate the NLRP3 inflammasome in macrophages and contribute to HSC activation. Furthermore, ER stress and mitochondrial-derived ROS activate apoptosis signal-regulating kinase 1 (ASK1), which promotes HSC activation and drives liver fibrosis. ASK1 inhibition has been shown to improve hepatic inflammation and fibrosis. WAT, white adipose tissue; BAT, brown adipose tissue; KHK, ketohexokinase; G6P, glucose-6-phosphate; F6P, fructose-6-phosphate; TAG, triacylglycerol; DGAT2, diacylglycerol O-acyltransferase 2; DAG, diacylglycerol; UFA, unsaturated fatty acids; SCD1, stearoyl-CoA desaturase 1; FAS, fatty acid synthase; ACC, acetyl-CoA carboxylase; HMG-CoA, 3-hydroxy-3-methylglutaryl–CoA; HMGCR, 3-hydroxy-3-methylglutaryl–CoA reductase; SPT, serine palmitoyltransferase; ACSS2, acetyl-CoA synthetase.

Most of the hepatic fat accumulation in MASLD originates from white adipose tissue, particularly through increased flux of non-esterified fatty acids (NEFAs) (Figure 1). In healthy subjects, postprandial insulin release rapidly suppresses lipolysis and hepatic NEFA delivery; however, adipose insulin resistance blunts this suppression, such that NEFA release continues at high rates despite elevated insulin levels (16, 55–57). Upper body subcutaneous and visceral adipose tissues are the most important contributors to circulating NEFA reaching the liver (58, 59), and while the visceral adipose tissue mass is much smaller, it drains directly into the portal vein, potentially explaining the strong correlation between visceral fat and liver triglycerides (60, 61), a relationship also observed in lean individuals (62). In children and adolescents, prospective changes in insulin-mediated suppression of NEFA levels after a meal are associated with ALT levels (63), and in adults, adipose tissue insulin resistance and lipolysis are directly correlated with increasing severity of MASH (64). Interestingly, an important driver of this relationship appears to be markers of adipose tissue fibrosis (65), an effect that may involve the release of endotrophin (66, 67). Importantly, numerous clinical trials with different PPAR (68–71) and GLP-1 receptor agonists (60, 72–74) support the concept that reducing adipose tissue insulin resistance and lipolysis has favorable effects on MASH and fibrosis (Figure 3).

Brown adipose tissue (BAT) could also potentially play a role in the development of MASH. Cold exposure in mice and humans increases oxidation of fatty acids from intracellular triglyceride lipolysis within BAT, and this leads to enhanced energy expenditure and heat production (75). In rodents, activated BAT also plays a major role in the clearance of circulating triglycerides (76); however, in humans, even with sustained cold-induced metabolic activation, BAT contributes less than 1% to systemic clearance of chylomicron fatty acids (77) and circulating NEFA (78). Similarly, while beige adipocytes (with intermediate thermogenic activity between white and brown adipocytes) have a high metabolic activity in rodents, in humans they do not contribute to whole-body energy expenditure (79). These data suggest that in humans BAT thermogenesis is unlikely to protect against lipid overload. However, studies in adults and children have shown a negative relationship between the abundance of BAT and liver steatosis, independent of obesity, suggesting that additional mechanisms may be important (80–82).

An emerging mechanism linking white and brown adipose tissue with MASH may involve the branched-chain amino acids (BCAAs) leucine, isoleucine, and valine (83–86). Both white and brown adipose tissue were shown to act as sinks protecting the liver from BCAA effects, which were independent of their thermogenic activity (87). Elevated BCAAs are a major carbon source for liver DNL (discussed in detail below) (86, 88), activating a central enzyme in the pathway, ATP-citrate lyase (ACLY) (89). In addition to promoting DNL, BCAAs also promote a shift toward glycolysis and a proinflammatory phenotype in monocyte-derived macrophages, leading to increases in reactive oxygen species (ROS) and cytokine production that accelerated liver fibrosis in *db/db* mice, a model of diabetes (90). Consistent with the potential importance of the adipose-BCAA-liver axis, the GLP-1/GIP receptor agonist tirzepatide, which reduced MASH in phase II studies (72), also improved adipose tissue insulin sensitivity and lowered circulating BCAA (91), effects that in mice are associated with increased BCAA metabolism within BAT (92). Similarly, bariatric surgeries (also highly effective for reducing MASH; ref. 93) were found to lower BCAAs in mice, through pathways that were independent of GLP-1 but required FGF-21 (94). BCAAs were also reduced in people with MASH who were treated with an inhibitor of the mitochondrial pyruvate carrier (95). These data suggest that metabolically based therapies associated with improvements in adipose tissue insulin sensitivity such as those targeting GLP-1 receptors, PPARs, and FGF-21 may not only reduce steatosis by reducing NEFA flux but may also potentially exert antifibrotic effects by inhibiting endotrophin and BCAAs (Figure 3). Future studies investigating these pathways will be important to better understand which populations may benefit most from these therapies.

## Mechanisms regulating DNL

The generation of fatty acids from glucose, fructose, and other substrates, including lactate, acetate, and amino acids, is mediated through DNL (96, 97) (Figure 3). DNL is elevated in people with MASLD and MASH (98–100) and is initiated when excess substrate availability from the oxidation of fatty acids, carbohydrates, and amino acids converges at the first step of the tricarboxylic acid (TCA) cycle, leading to increases in mitochondrial citrate (101). As the TCA cycle cannot store metabolic intermediates, in the absence of an energetic sink (i.e., ATP demand), citrate is exported from the mitochondria into the cytosol by the citrate isotransporter (CIC/SLC25A1) (96). Once in the cytosol, citrate is converted to acetyl-CoA and oxaloacetate by ACLY. Acetyl-CoA is also used to generate cholesterol via the mevalonate pathway, and inhibition of ACLY by bempedoic acid leads to increases in the LDL receptor in the liver and reductions in LDL-cholesterol and atherosclerosis (102). Acetyl-CoA can also enter the nucleus, where it influences histone acetylation and gene expression, therefore linking substrate supply with transcriptional control (103). However, the majority of cytosolic acetyl-CoA is converted to malonyl-CoA in the first committed step of the DNL pathway by acetyl-CoA carboxylase (ACC). Malonyl-CoA also inhibits the activity of carnitine palmitoyl-transferase-1 (CPT1), the rate-limiting enzyme controlling entry of fatty acyl-CoA to the mitochondria and fatty acid oxidation; thus DNL and fatty acid oxidation are usually inversely regulated (104). ACC exists as two distinct isoforms in the liver, ACC1 and ACC2, and while dogma suggests they have distinct functions controlling DNL and fatty acid oxidation, respectively, it is now recognized that they have overlapping roles and that inhibition of both isoforms is necessary to maximize therapeutic responses for MASLD and MASH (105, 106). Importantly, DNL remains elevated in people with MASH and cirrhosis despite reductions in steatosis, suggesting that it is a key driver of disease development (107).

Among dietary nutrients, fructose (a component of table sugar and high-fructose corn syrup) is a particularly potent inducer of hepatic DNL (reviewed in ref. 108). Fructose and metabolic intermediates generated by the gut microbiome, e.g., acetate, bypass normal insulin-regulated checkpoints of glycolysis, leading to the accumulation of acetyl-CoA (108, 109) (Figure 3). At the same time, fructose and its metabolites, like glucose-6-phosphate and xylulose-5-phosphate, activate carbohydrate-responsive element–binding protein (ChREBP) and sterol regulatory element–binding protein 1c (SREBP-1c), regulators of transcriptional programs that increase the expression of DNL enzymes (108). Lastly, fructose-induced endotoxemia activates MyD88-mediated inflammatory processes in liver myeloid cells, increasing TNF (110). TNF suppresses AMP-activated protein kinase (AMPK), leading to reduced phosphorylation of ACC (111), which increases liver DNL, steatosis, inflammation, and fibrosis (106). In people with MASLD, fructose has a much greater DNL-stimulating effect than glucose (112–114). Pharmacological inhibition of ketohexokinase, the first step in the metabolism of fructose, lowers liver ChREBP, hyperinsulinemia, hypertriglyceridemia, and hepatic steatosis in mice fed a Western diet (115, 116) while also reducing liver steatosis, serum uric acid, and high-sensitivity CRP in people with MASLD (117). However, this reduction in steatosis of about 25% was relatively modest, which may be related to the multiple substrates feeding into DNL, including elevations in gut microbiome–derived ethanol (118), which would be converted in the liver to acetate and acetyl-CoA via acetyl-CoA synthetase 2 (ACSS2) (109), thus bypassing regulation via ketohexokinase. These findings suggest that while targeting fructose metabolism can reduce hepatic steatosis, its overall impact may be limited by alternative substrates that continue to drive DNL through bypass pathways such as ACSS2.

In addition to carbohydrates such as fructose, fatty acids derived from insulin-resistant adipose tissue are also an important factor contributing to elevations in DNL in MASLD and MASH (114, 119). Mechanistically, increased adipose tissue–derived free fatty acids delivered to the liver allosterically activate AMPK, leading to increases in fatty acid oxidation (120, 121), which leads to generation of acetyl-CoA (122) independently of ACLY (86). Highlighting the important contribution and overlap between adipose tissue insulin resistance and DNL are recent findings showing that in people with MASLD, DNL accounts for approximately 40% of total triglyceride accretion during the postprandial period (119). These data suggest that, in contrast to previous studies that were done under fasting conditions (98–100), DNL is the major contributing factor to steatosis, and indicate the critical and interrelated role for adipose tissue insulin resistance in priming the DNL pathway (Figure 1).

## DNL inhibitors and ceramides

Highlighting the critical role of DNL in MASH are findings with pharmacological inhibitors of cytosolic citrate (123, 124), ACLY (125), ACC (126, 127), or fatty acid synthase (FAS) (128, 129), which exert favorable activities on reducing DNL and lowering steatosis in preclinical models and, in some cases, clinical populations (reviewed in refs. 96, 130) (Figure 3). Inhibition of DNL can also be achieved indirectly by inhibition of distal steps in triglyceride synthesis, including stearoyl-CoA desaturase 1 (SCD1) (131) and diacylglycerol acyltransferase 2 (DGAT2) (132–135); recent studies with DGAT2 inhibitors have revealed that this is due to increases in endoplasmic reticulum phosphatidylethanolamine, which blocks cleavage and activation of SREBP-1c (136). In addition to inhibiting steatosis, blockade of ACC (137), ACLY (125), FAS (128), or SCD1 (138) or activation of AMPK (which inhibits ACC) (139) also reduces TGF-β–induced activation of HSCs. In liver macrophages, activation of AMPK (140) or inhibition of ACLY (141) or ACC (142) also reduces liver inflammation in mouse models. These actions on multiple cell types within the liver may explain in part the discordant findings between beneficial effects with pharmacological inhibitors and the benign or detrimental findings with hepatocyte-selective genetic inhibition of these targets (109, 143–145). Despite the positive effects of ACC and FAS inhibitors to reduce MASH, they also increase serum triglycerides by increasing liver VLDL production and/or potentially clearance (126, 127, 129), effects that can be blocked by inhibition of DGAT2 (146, 147). These effects are not observed with AMPK activators (139), the dual AMPK activator/ACLY inhibitor bempedoic acid (102), or the SCD1 inhibitor/AMPK activator Aramchol (131).

An important mechanism linking inhibition of DNL with reductions in inflammation and fibrosis are ceramides, which are preferentially synthesized via this pathway in MASH (126, 127, 148). Recent studies using spatial lipidomics integrated with transcriptomics and imaging have revealed that sphingolipid metabolism is dysregulated in liver fibrosis and closely associated with myofibroblast-rich regions in both humans and mouse models of MASH (149). In mice fed a Western diet with glucose in the drinking water (i.e., the DIAMOND model), loss-of-function mutations in *Pnpla3* increase liver ceramide and genetic signatures linked to elevations in ceramide synthesis, STAT3, and both innate and adaptive immune-inflammatory pathways (150). In macrophages, ceramides activate the NLRP3 inflammasome, leading to caspase-1 activation and the release of IL-1β and IL-18, which propagate liver inflammation and fibrosis (151), effects that may involve direct binding to the G_q_ receptors CYSLTR2 and P2RY6 (152). Similarly, the expression of acid ceramidases, which break down ceramide into sphingosine and fatty acids, is correlated with MASH and fibrosis, and acid ceramidase inhibition leads to greater fibrosis through activation of YAP/TAZ (153, 154). Pharmacological inhibition of ceramide synthesis, by suppression of serine palmitoyltransferase (SPT) using myriocin (155, 156), CH5169356 (157), or adiponectin (which also activates PPARγ) (158), suppresses HSC activation and attenuates fibrosis in preclinical models. These data suggest that inhibition of DNL may exert dual therapeutic benefits in MASH by lowering both steatosis and ceramide-driven paracrine signaling that links hepatocyte injury to chronic immune and fibrotic activation.

Given that DNL is upregulated across all stages of MASLD, including MASH and cirrhosis, these data suggest that inhibiting enzymes critical for the control of DNL may exert favorable effects across the disease spectrum by not only reducing steatosis but also inhibiting the activation of HSCs. However, given the metabolic flexibility and substantial redundancy in the pathway, therapies that inhibit multiple enzymes may be necessary to maximize efficacy and avoid increases in atherogenic risk profiles.

## Impaired hepatic fatty acid oxidation and mitochondrial dysfunction

Liver fatty acid oxidation is critical for generating ATP and reducing equivalents required for gluconeogenesis, DNL, and ketone body and lipid droplet production as well VLDL packaging. The liver oxidizes approximately 35% of circulating NEFA over 6 hours after a meal (16), making impaired fatty acid oxidation an important mechanism contributing to MASLD (159).

**Figure 4:**
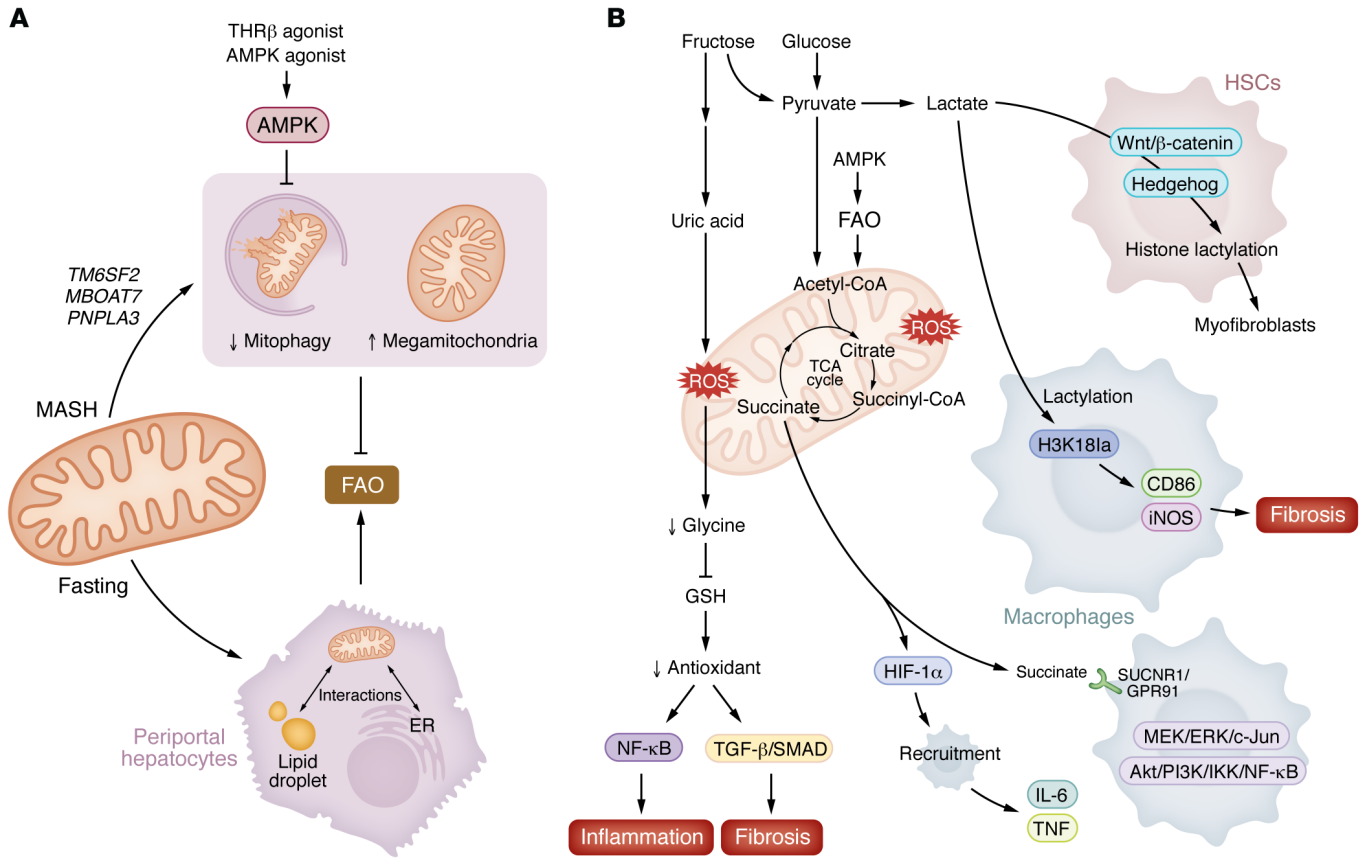
Impaired FAO and mitochondrial dysfunction in MASH. (**A**) Megamitochondria are a hallmark of MASH. Impaired mitophagy contributes to mitochondrial structural abnormalities in the livers of individuals with MASH, leading to reduced FAO. Fasting promotes interactions between the ER and mitochondria in periportal hepatocytes. Fasting also enhances mitochondria–lipid droplet interactions, which are increased during early MASLD but decline as fibrosis progresses. Genetic variants, such as *TM6SF2*, *MBOAT7*, and *PNPLA3*, promote mitochondrial dysfunction, which is characterized by swollen, fragmented cristae, and impaired bioenergetic capacity. (**B**) Dysfunctional mitochondria exacerbate liver injury by generating incomplete oxidation byproducts, including ROS, lactate, and succinate. Fructose metabolism further contributes to mitochondrial oxidative stress by generating uric acid, which depletes glycine and lowers levels of glutathione (GSH), the primary cellular antioxidant responsible for maintaining redox homeostasis. This oxidative imbalance activates proinflammatory and fibrogenic signaling pathways, including NF-κB and TGF-β/SMAD. A key feature of impaired oxidative phosphorylation is elevated lactate production. In Kupffer cells and monocyte-derived macrophages, lactate induces histone lactylation at H3K18la, upregulating fibrogenic genes such as CD86 and iNOS. In HSCs, activation via hedgehog or Wnt/β-catenin signaling enhances lactate utilization to fuel differentiation into myofibroblasts. The TCA cycle intermediate succinate binds to succinate receptor 1 (SUCNR1/GPR91), activating MEK/ER/c-Jun and PI3K/Akt/IKK/NF-κB pathways to promote inflammation. Succinate also stabilizes hypoxia-inducible factor 1α (HIF-1α), stimulating macrophage recruitment and the production of proinflammatory cytokines, including IL-6 and TNF.

Stable isotope studies have found that hepatic fatty acid oxidation rates are unaltered (160) or can be elevated by 30%–50% in individuals with MASLD compared with controls (161–164), suggesting this is unlikely to be the cause of steatosis. However, rates of fatty acid oxidation do appear to decline with advancement to MASH (161, 165). Similar findings using a noninvasive ^13^C-palmitate breath test have also found that individuals with MASLD oxidized approximately 27% less of an orally administered fatty acid load than healthy individuals (166). In mice, MASLD is associated with increased whole-liver fatty acid oxidative metabolism (assessed using deuterium magnetic resonance imaging) but with reduced ATP, citrate synthase, and fatty acid oxidation when expressed relative to the weight of the liver (167). A similar reduction in fatty acid oxidation is observed in isolated liver slices in people with MASH compared with those with obesity and MASLD (160, 168). Consistent with impairments in liver fatty acid oxidation being important in MASH, increasing mitochondrial uncoupling reduces hepatic steatosis in clinical trials (169). And while the beneficial effects of mitochondrial uncouplers are commonly ascribed to increases in fatty acid oxidation, it is important to note that these compounds also reduce the reductive force and ATP required for DNL (97, 170), suggesting that this may be a more important mechanism for lowering steatosis in individuals with MASLD but not MASH. Taken together, these data suggest that during early stages of MASLD there may be increases in fatty acid oxidation, but with MASH, reductions in fatty acid oxidation per gram of tissue may occur (Figure 4).

Complicating interpretation of findings on fatty acid oxidation are emerging observations using spatially resolved metabolomics and proteomics that there may also be zone-specific shifts in intercellular mitochondrial activity (171). For example, studies have found that mitochondria from periportal hepatocytes have a higher capacity for fatty acid oxidation than those from pericentral hepatocytes (172). These zonal shifts in periportal mitochondrial metabolism correlate with increases in AMPK phosphorylation of ACC (172), which is critical for fatty acid oxidation in the liver (106). Consistent with increases in fatty acid oxidation and activation of AMPK, fasting induces interactions between the endoplasmic reticulum and mitochondria in periportal hepatocytes but not in pericentral hepatocytes, effects that are blunted in obesity (173). Fasting also promotes interactions between perilipid droplets and mitochondria (174), a relationship that is increased in early stages of MASLD but then declines with progression of fibrosis in the choline-deficient high-fat diet mouse model of MASH (175). These findings highlight the emerging importance of hepatic zonation in regulating mitochondrial fatty acid oxidation and suggest that disruptions to periportal mitochondrial dynamics may contribute to the metabolic dysfunction observed in MASH.

Reductions in mitochondrial function in MASH have been associated with megamitochondria and disorganized and fragmented cristae (165, 176). These mitochondrial derangements are consistent with reductions in a process called mitophagy, the selective degradation of mitochondria via autophagy, that is reduced in livers of people with MASH (168). Genetic variants linked with MASH including *TM6SF2* and *MBOAT7* promote swollen and fragmented cristae when overexpressed in cultured cells (177), while individuals with mutations in *PNPLA3* also have impaired mitochondrial function (178) (Figure 4). In the setting of high-fat diet, a mouse model expressing a phospho-deficient point mutation in AMPK, leading to reduced AMPK activity in liver, exhibited increased MASH and megamitochondria with disordered cristae that were associated with impaired fatty acid oxidation and mitophagy (121). In contrast, an AMPK activator that binds to the same residue on AMPK reduced steatosis, inflammation, and fibrosis in mice (139) and reduced steatosis in people with type 2 diabetes (179). Interestingly, thyroid receptor β (THRβ) agonists have been shown to also activate AMPK and increase mitophagy (180). Future studies investigating whether liver AMPK is important for mediating the beneficial effects of the recently approved THRβ agonist resmetirom in reducing MASH and fibrosis (181) will be important for understanding the therapeutic importance of this pathway.

## Incomplete oxidative by-products link mitochondria, inflammation, and fibrosis

In addition to having a reduced capacity for oxidizing fatty acids, dysfunctional mitochondria also perpetuate hepatic injury by generating incomplete oxidation by-products including ROS, lactate, and succinate, which in turn activate macrophages and HSCs (Figure 4). Steatotic hepatocytes exhibit evidence of chronic oxidative stress including high levels of mitochondrial hydrogen peroxide and lipid peroxidation by-products (168, 176). Oxidation of fructose also generates uric acid, which contributes to mitochondrial oxidative stress (182). Increases in oxidative stress deplete glycine, which is important for the synthesis of glutathione (GSH), the main cellular antioxidant that maintains intracellular redox status. Treatment of mice and nonhuman primates with DT-109, which stimulates de novo glutathione synthesis, restores hepatic fatty acid oxidation, while suppressing inflammation and fibrosis, effects associated with the suppression of NF-κB target genes and TGF-β/SMAD signaling, respectively (183–185). Similarly, treatment of mice with the SCD1 inhibitor Aramchol increases glutathione and fatty acid oxidation (186). These data suggest that restoration of glutathione metabolism may have positive effects on reducing inflammation and fibrosis by enhancing mitochondrial fatty acid oxidation.

Another hallmark of impaired oxidative phosphorylation is an increase in lactate. In Kupffer cells and monocyte-derived macrophages, lactate has been shown to upregulate genes associated with tissue remodeling and fibrosis including CD86 and iNOS, effects that are mediated through histone lactylation of H3K18la, an epigenetic modification that directly stimulates gene transcription from chromatin (187). HSCs activated by Hedgehog or Wnt/β-catenin signaling (188) utilize lactate as a fuel source to promote their differentiation into myofibroblasts (189), an effect that also involves histone lactylation (190) and stabilization of hypoxia-inducible factor-1α (HIF-1α) (191). Importantly, therapies that directly target either HSC and/or macrophage lactate uptake (by inhibiting MCT1 [ref. 189]) or lactate production (by blocking lactate dehydrogenase [ref. 192] or hexokinase 2 [refs. 187, 193]) reduce inflammation and fibrosis in mouse models of MASH. Thus, manipulation of lactate serves as a critical metabolic bridge between hepatocyte mitochondrial function immune activation and HSC-mediated fibrosis; however, therapies must be specifically tailored to HSCs or macrophages, since inhibiting uptake in hepatocytes may accelerate inflammation and fibrosis (189).

Lastly, an emerging regulator of inflammation and fibrosis in MASH is the TCA cycle metabolic intermediate succinate. Liver and serum succinate are increased in MASLD and MASH (194). While increases in liver succinate are thought to be derived primarily from hepatocytes, increases in succinate may also be related to reductions in BAT (195). Succinate receptor 1 (SUCNR1/GPR91) is expressed in both liver macrophages and HSCs and leads to dissociation of the Gα subunit from the Gβγ dimer, which activates MEK/ERK/c-Jun and Akt/PI3K/IKK/NF-κB signaling (196, 197). Succinate also promotes inflammation and macrophage recruitment through stabilization of HIF-1α, leading to production of proinflammatory cytokines such as IL-6 and TNF (195). In HSCs, succinate activates fibrotic remodeling in rodent models, promoting HSC activation, proliferation, and migration (194, 196, 198, 199). Interestingly, consistent with a role for succinate-mediated immune activation and HSC fibrogenesis, treatment with an FGF-21 analog (LY2405319) in the methionine/choline-deficient (MCD) diet model of MASH reduces succinate accumulation in the liver and serum, findings that correspond with reductions in HSC activation and inflammation (197). Future studies investigating whether the beneficial effects of FGF-21 analogs in people with advanced fibrosis and cirrhosis are also correlated with reductions in succinate may be important for identifying the mechanisms by which these therapies resolve inflammation and fibrosis independently of their potential metabolic actions on adipocyte insulin sensitivity.

## Conclusion

The development of MASH reflects a breakdown in metabolic coordination between hepatocytes, macrophages, and HSCs. Excess fatty acid delivery from the diet and insulin-resistant adipose tissue, combined with elevated lipogenesis and impaired mitochondrial oxidation, leads to the buildup of metabolites like cholesterol, ceramides, lactate, and succinate. These intermediates trigger stress and inflammatory pathways in hepatocytes and immune cells, while also promoting stellate cell activation and matrix production. Rather than acting independently, these signals converge across cell types, reinforcing a cycle of metabolic dysfunction, immune activation, and fibrosis, highlighting numerous potential therapeutic targets for MASLD/MASH (Figure 3). Spatial shifts in mitochondrial activity and nutrient handling further amplify this response as the disease progresses.

Despite substantial progress with multiple therapies in phase III clinical trials for MASH targeting diverse pathways, many questions remain about how current therapies reduce MASH and fibrosis, and why only a subset of individuals respond (Figure 3). Agents such as THRβ, GLP-1 receptor, FGF-21 receptor, and PPAR agonists clearly reduce liver fat, but their effects on inflammation, fibrosis, and crosstalk between hepatocytes, macrophages, and HSCs remain incompletely understood. One critical area that requires further investigation is how these therapies influence the handling of specific fatty acid species. Saturated, monounsaturated, and polyunsaturated fatty acids have distinct metabolic fates and biological effects, yet most studies have relied on palmitate tracers to assess lipid flux. Stable isotope labeling with additional tracers, combined with spatial imaging and metabolomic approaches, will be needed to determine how these therapies impact cell type–specific lipid metabolism and intercellular signaling. In particular, understanding how they affect mitochondrial function, including oxidation, redox balance, and incomplete metabolite production, in distinct hepatic zones may help explain differential responses and guide therapeutic optimization. In parallel, designing small molecules that act on multiple metabolic targets may be necessary to effectively disrupt the feed-forward loops driving disease progression. Machine learning–based approaches, such as those recently used to develop dual ACC and DGAT inhibitors (200), offer promising strategies to target complex metabolic networks in a more integrated and personalized manner. Ultimately, combining mechanistic insight with a new generation of precision tools will be essential to move beyond liver fat and toward therapies that durably resolve inflammation and fibrosis.

## Version 1 09/16/2025

Electronic publication
